# Isolated Digital Dupuytren’s Cord of the Finger: Correlative MRI and Ultrasound Findings

**DOI:** 10.5334/jbsr.4192

**Published:** 2026-02-06

**Authors:** Caroline Chabot, Lokmane Taihi

**Affiliations:** 1 Department of Radiology, Institut de Recherche Expérimentale et Clinique (IREC), Cliniques Universitaires Saint Luc, Université Catholique de Louvain, Brussels, Belgium

**Keywords:** digital cord, Dupuytren’s disease, fibromatosis, MRI, ultrasound

## Abstract

*Teaching point:* Combined MRI and ultrasound allow accurate identification of isolated digital Dupuytren’s cords, thereby supporting diagnosis and surgical planning.

## Clinical History

**Figure 1:**
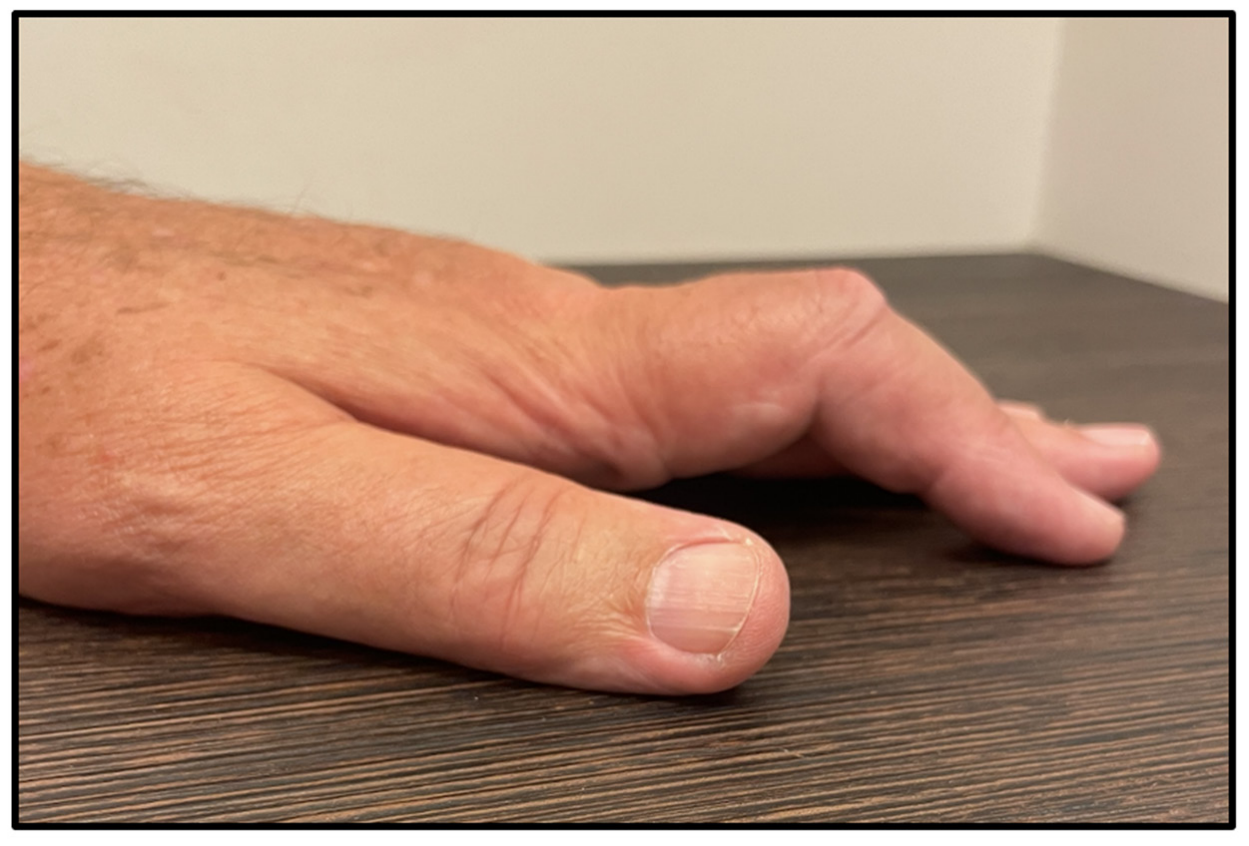
Mild pseudo-boutonnière appearance of the index finger due to progressive stiffness and subtle digital deformity.

A 71-year-old man presented with a several-month history of progressive stiffness of the index finger, without pain, trauma, or prior hand surgery. Physical examination revealed subtle focal skin retraction on the radial–palmar aspect of the proximal phalanx, associated with a fixed flexion contracture of the proximal interphalangeal (PIP) joint and a mild pseudo-boutonnière appearance (Figure 1).

**Figure 2:**
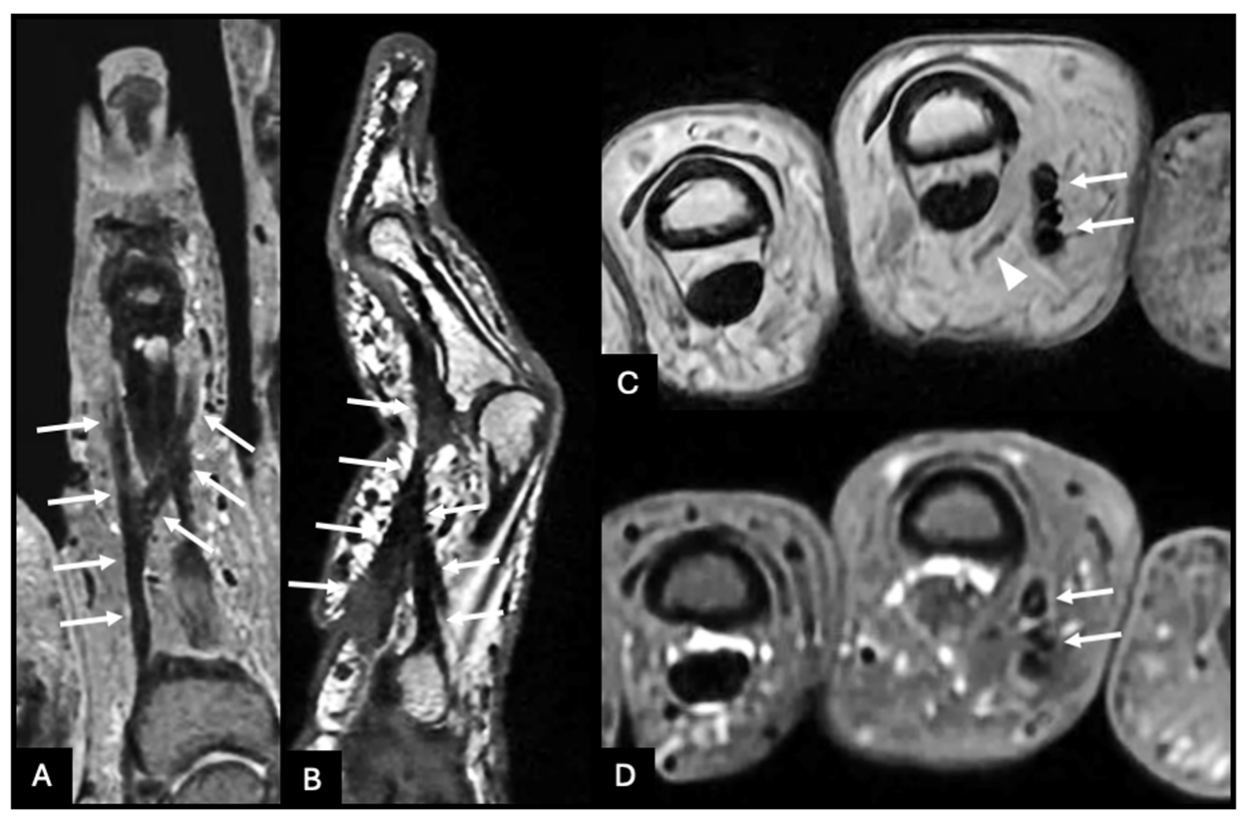
Magnetic resonance imaging showing a hypodermal fibrous cord along the radial and palmar aspects of the proximal phalanx (arrows), bifurcating distally into two slips inserting on the base of the middle phalanx. The radial neurovascular bundle is displaced in a characteristic pattern without encasement (arrowhead).

MRI demonstrated a hypodermal fibrous cord along the radial and palmar aspects of the proximal phalanx. Proximally, the cord originated from the radial base of P1, causing focal skin tethering. Distally, it bifurcated into two slips inserting on the radial and ulnar aspects of the base of P2, coursing on either side of the flexor tendon apparatus. A mild displacement of the radial neurovascular bundle was observed, without encasement or invasion (Figure 2, arrows).

**Figure 3:**
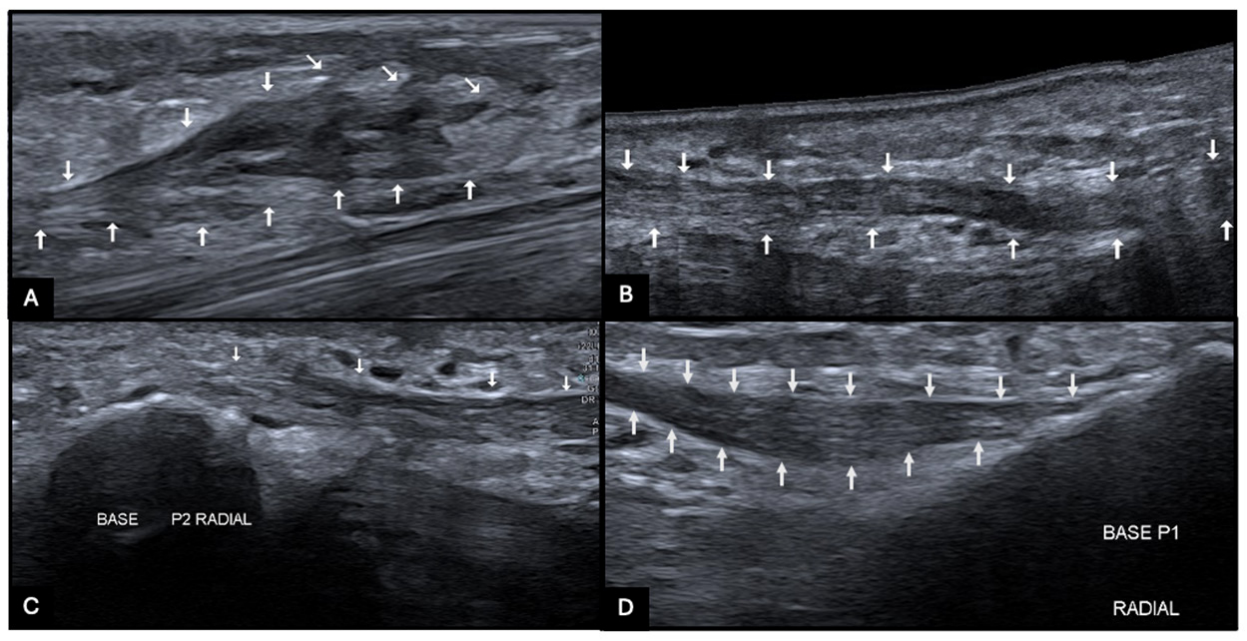
Ultrasound showing a superficial hyperechoic digital cord (arrows).

Ultrasound confirmed a superficial hyperechoic, fibrillary cord (Figure 3, arrows) with preserved flexor tendon mobility and dynamic neurovascular bundle displacement during finger motion.

These imaging findings were diagnostic of an isolated digital Dupuytren’s cord. Given the progressive functional impairment and the development of a pseudo-boutonnière posture, surgical excision was performed.

## Comments

Isolated digital Dupuytren’s cord is an under-recognized variant of Dupuytren’s disease confined to the finger. It represents localized fibromatosis of the digital fascial system and may produce progressive stiffness despite minimal early clinical signs.

This case illustrates the typical morphology and evolution of a digital cord. Such cords generally arise from the base of the proximal phalanx and follow an oblique hypodermal course toward the middle phalanx, occasionally bifurcating distally. Their relationship with surrounding structures, including tendons and adjacent soft tissues, explains the potential for progressive PIP joint contracture.

Clinically, digital cords may be subtle because of limited early skin adherence and minimal deformity, which can delay recognition. They may affect any digit and are frequently associated with other fibroproliferative manifestations within the hand [1].

From an imaging standpoint, MRI allows a comprehensive evaluation of cord morphology, extent, and insertion sites, while ultrasound provides high-resolution assessment of superficial fibrous tissue and enables dynamic evaluation during finger motion. The combined use of these modalities improves diagnostic confidence and helps anticipate functional progression.

This case highlights the importance for radiologists of recognizing the imaging appearance of isolated digital Dupuytren’s disease to facilitate timely diagnosis and appropriate management.
